# Trophoblast Cell Surface Antigen 2 (Trop-2) Protein is Highly Expressed in Salivary Gland Carcinomas and Represents a Potential Therapeutic Target

**DOI:** 10.1007/s12105-021-01325-5

**Published:** 2021-04-22

**Authors:** Philipp Wolber, Lisa Nachtsheim, Franziska Hoffmann, Jens Peter Klußmann, Moritz Meyer, Ferdinand von Eggeling, Orlando Guntinas-Lichius, Alexander Quaas, Christoph Arolt

**Affiliations:** 1 Department of Otorhinolaryngology, Head and Neck Surgery, University of Cologne, Medical Faculty, Cologne, Germany; 2 Department of Otorhinolaryngology, Head and Neck Surgery, Jena University Hospital, Friedrich-Schiller-University Jena, Jena, Germany; 3 Department of Otorhinolaryngology, MALDI Imaging and Innovative Biophotonics, Jena University Hospital, Friedrich-Schiller-University Jena, Jena, Germany; 4 Center for Molecular Medicine Cologne (CMMC), University of Cologne, Faculty of Medicine and University Hospital Cologne, Cologne, Germany; 5 Department of Otorhinolaryngology, Head and Neck Surgery, University Hospital Essen, University Duisburg-Essen, Essen, Germany; 6 DFG Core Unit Jena Biophotonic and Imaging Laboratory (JBIL), MALDI Imaging, Core Unit Proteome Analysis, Jena University Hospital, Friedrich-Schiller-University Jena, Jena, Germany; 7 Department of Pathology, University of Cologne, Medical Faculty, Cologne, Germany

**Keywords:** Salivary gland neoplasms, Immunohistochemistry, Molecular targeted therapy, Retrospective studies, Immunoconjugates, Spectrometry, Mass, Matrix-assisted laser desorption/ionization

## Abstract

Treatment options for unresectable, recurrent or metastatic salivary gland carcinomas (SGC) are scarce. Trophoblast cell surface antigen 2 (Trop-2) is a transmembrane glycoprotein that is involved in a variety of oncogenic cell signaling pathways. Its potential as a target for the antibody–drug conjugate sacituzumab govitecan has already been demonstrated in different tumor entities. The United States Food and Drug Administration approved this antibody–drug conjugate for the treatment of metastatic triple-negative breast cancer. Here, we aimed to investigate Trop-2 protein expression in different entities of SGCs. We retrospectively reviewed the medical records of all patients that underwent surgery for a primary SGC in a tertiary referral center between 1990 and 2014. Immunohistochemical (IHC) staining for Trop-2 was performed and rated as negative, weak, moderate or high using a semiquantitative score. Additionally, representative cases were analyzed using MALDI-mass spectrometry (MS) imaging to confirm the IHC results. The cohort consisted of 114 tumors of the parotid gland (90.4%) and submandibular gland (9.6%). It mainly included mucoepidermoid, salivary duct and adenoid cystic carcinomas. In IHC samples, 44% showed high, 38% moderate and 10% weak expression rates of Trop-2. MALDI-MS imaging confirmed the presence of Trop-2 protein in 80% of the tested tumor samples. This is the first study to demonstrate that several types of SGC express Trop-2 with variable intensity. Since there are currently few systemic treatment options for advanced SGCs, Trop-2 represents a promising target for further clinical studies, for instance, with sacituzumab govitecan.

## Introduction

Salivary gland carcinomas (SGC) consist of a heterogeneous group of more than a dozen tumors. Although each entity displays recurrent genomic alterations, significant heterogeneity can be found within each group [1, 2]. The majority of early stage tumors (T1-2) can be treated by tumor resection. Additionally, performance of a neck dissection is recommended in high grade carcinomas even in the absence of clinical or radiological evidence of locoregional metastatic nodal spread and in low-grade tumors with suspicion of (loco-regional) nodal involvement [3]. Generally, in advanced-stage tumors (T3-4), high-grade tumors, positive or close margins or in the presence of lymph node metastasis, vascular and perineural invasion, adjuvant radiation therapy is indicated [4, 5]. For adenoid cystic carcinoma and other high-risk pathologies (e.g., high-grade mucoepidermoid carcinoma, salivary duct carcinoma), especially those arising in anatomic sites not amenable to wide resection, such as the nasal cavity and paranasal sinuses, many authors recommend adjuvant radiation therapy to increase local control rates even after complete resection due to the tumors’ aggressive and often perineural growth [4, 6–10]. However, the role of adjuvant radiation therapy in early-stage tumors without adverse features such as positive margins, perineural invasion and negative neck nodes is contentious, as some authors failed to confirm adjuvant radiation therapy as a prognostic factor [11, 12].

Definitive treatment regimens without surgery consisting of combined chemoradiotherapy with curative intent only come into consideration for patients unable to undergo surgery or for unresectable cancers, such as with skull base involvement [13, 14]. In rare cases, chemotherapy alone can be applied in a neoadjuvant setting to reduce tumor volume for subsequent salvage surgery, but does not play a role in the current standard curative treatment of primary SGC [15–17]. In case of unresectable, recurrent or metastatic cancer, mainly platinum-based palliative chemotherapy regimens are applied. However, randomized controlled trials are lacking and toxicity is considerable [18]. Currently, more information is gathered about possible molecular targets for a tailored therapy for SGCs [19, 20]. Promising results have been achieved with, for example, Herceptin and NTRK inhibitors in selected patients with primarily unresectable disease [10, 21, 22].

Another interesting target is trophoblast cell surface antigen 2 (Trop-2) which is a transmembrane glycoprotein that is involved in a variety of cell signaling pathways including proliferation, survival, self-renewal, and invasion. Cell signaling mediated by Trop-2 involves the expression of Ki-67, activation of MAPK signaling and cell cycle progression by increasing levels of cyclin D1. Trop-2 expression can induce transcription factor AP-1 leading to upregulation of carcinogenesis associated genes [17, 23]. Expression of Trop-2 is associated with poor outcome, particularly in solid tumors [24]. The potential of Trop-2 as a target for combination therapy using the antibody–drug conjugate sacituzumab govitecan, has already been demonstrated in a number of different tumor entities, such as urothelial carcinoma, squamous cell lung carcinoma and breast cancer [23, 25, 26]. In metastatic triple-negative breast cancer, the FDA has already approved this antibody–drug conjugate [26]. Studies indicate that sacituzumab govitecan mediates anti-tumor responses with varying expression of the Trop-2 protein [27, 28]. Until now, the relevance and distribution of Trop-2 protein expression in SGC has not been addressed (Table 1).

The aim of the current study was to test for the extent, distribution and intensity of Trop-2 protein expression in different entities of SGCs using a bimodal approach of immunohistochemistry and matrix-assisted laser desorption/ionization mass spectrometry (MALDI-MS) imaging.

## Materials and Methods

### Patients

We searched the medical records of all patients with SGCs that underwent primary surgery at the Department of Otorhinolaryngology, Head and Neck Surgery, University Hospital of Cologne, Germany between 1990 and 2014, including all patients with sufficient formalin-fixed paraffin-embedded (FFPE) material for further analysis. The study was performed according to the regulations of the Ethics Committee of the University of Cologne.

### Data Collection

Patients’ clinical records and histopathologic reports were reviewed with respect to tumor characteristics (stage of disease at the time of diagnosis according to AJCC TNM staging system (8th edition, 2020)) and patient characteristics.

### Immunohistochemistry for Trop-2

All histological diagnoses were reviewed by two pathologists with special expertise in the field (C.A. and A.Q). For tissue microarrays (TMAs), a self-constructed semi-automated precision instrument was used to punch out four tissue cylinders with a diameter of 1.2 mm from selected tumor tissue blocks. Subsequently, the cylinders were embedded in empty recipient paraffin blocks. From the resulting TMA blocks, sections of 4 μm were cut and transferred to an adhesive-coated slide system (Instrumedics Inc., Hackensack, NJ, USA). Immunohistochemistry was performed using a Leica BOND-MAX stainer (Leica Biosystems, Germany), according to the protocol. The anti-Trop2 rabbit IgG monoclonal antibody EPR20043 (Abcam, Cambridge, United Kingdom; dilution 1:1000) was used. After conjugation with an antibody-bound horseradish peroxidase, detection was carried out using Polymer Refine Detection Kit (Leica Biosystems, Germany). Counterstaining was done with hematoxylin and bluing reagent. As the cases in our cohort reflected the entire spectrum of membranous Trop-2 positivity, no reference specimens were used for immunoscore calibration. Several internationally established immunoscores take into account both the intensity of staining and the number of stained tumor cells. One of the more frequently used scores, the H-score, produces equivalent results as the semi-quantitative score that was used here [29]. Precisely, membranous basolateral or circumferential Trop-2 staining was assessed as follows: negative (< 1% Trop-2 expression of any intensity), weak (weak Trop-2 protein expression ≤ 70% or moderate protein expression ≤ 30%), moderate (weak Trop-2 protein expression > 70% or moderate expression 30–70% or strong expression ≤ 30%) or high (moderate Trop-2 protein expression > 70% or strong expression > 30%). In order to analyze a possible intra-tumoral heterogeneity of Trop-2 expression, 20 full sections were additionally stained (five negative cases and two, eight and five cases with weak, moderate and high expression, respectively). The scoring was independently performed by two pathologists with special interest in the field of salivary gland carcinomas (C.A. and A.Q.). In the rare case of a discrepant scoring result, a consensus score was agreed upon.

### MALDI-MS Imaging

For MALDI-MS Imaging, analysis sections consecutive to immunohistochemistry-stained sections were used. Three exemplary cases with high expression, two with moderate, three with weak and two with negative expression of Trop-2 in IHC were analyzed. Sample preparation including deparaffinization, antigen retrieval, tryptic digestion and matrix application was performed according to a previously published study [30]. MALDI-MS Imaging measurements were performed on ultrafleXtreme mass spectrometer (Bruker Daltonik GmbH, Germany). Data acquisition was operated in reflective negative mode with 50 µm spatial resolution (medium laser spot size) and 200 laser shots. For data and imaging analysis, SCiLS Lab software Version 2021a Premium 3D (Bruker Daltonik GmbH, Germany) was used. In silico protein digestion was performed using the ProteinProspector MS-Digest tool (Uniprot ID: P09758). For the generation of the box plots the mean intensity of all detected Trop-2 peptide masses per pixel were used. The m/z images of the TMA cores were generated as a fused image of all Trop-2 peptides. The expression of Trop-2 protein masses were evaluated with ROC analysis.

### Statistical Analysis

Analyses were performed using SPSS Statistics 26.0 (IBM, New York, NY, U.S.A.). Cross-tabulations, the Pearson’s χ^2^ and Fisher’s exact test were used to analyze interdependence of Trop-2 expression with clinical parameters. Kaplan–Meier method with 95% confidence intervals was used to test for disease free survival (DFS) probability rates. For this, statistical significance was tested by using the log-rank test. A p value < 0.05 was considered statistically significant.

## Results

### Demographics

We identified 114 patients, 59 females (51.8%) and 55 males (48.2%). The mean age was 55.8 ± 17.9 years (min. 16, max. 86 years) (Table).

### Tumor characteristics

103 tumors of the parotid gland (90.4%) and 11 tumors of the submandibular gland (9.6%) were identified including 24 (21.1%) mucoepidermoid carcinomas, 23 (20.2%) salivary duct carcinomas, 22 (19.3%) adenoid cystic carcinomas, 10 (8.8%) acinic cell carcinomas, nine (7.9%) adenocarcinomas not otherwise specified (NOS), seven (6.1%) epithelial myoepithelial carcinomas, seven (6.1%) secretory carcinomas, three (2.6%) basal cell adenocarcinomas, two (1.8%) carcinosarcomas, two (1.8%) myoepithelial carcinomas, two (1.8%) oncocytic cell carcinomas, one (0.9%) undifferentiated carcinoma, one (0.9%) carcinoma ex pleomorphic adenoma and one (0.9%) polymorphic adenocarcinoma. Additional clinical data is presented in the table.

### Expression of Trop-2

**Fig. 1:**
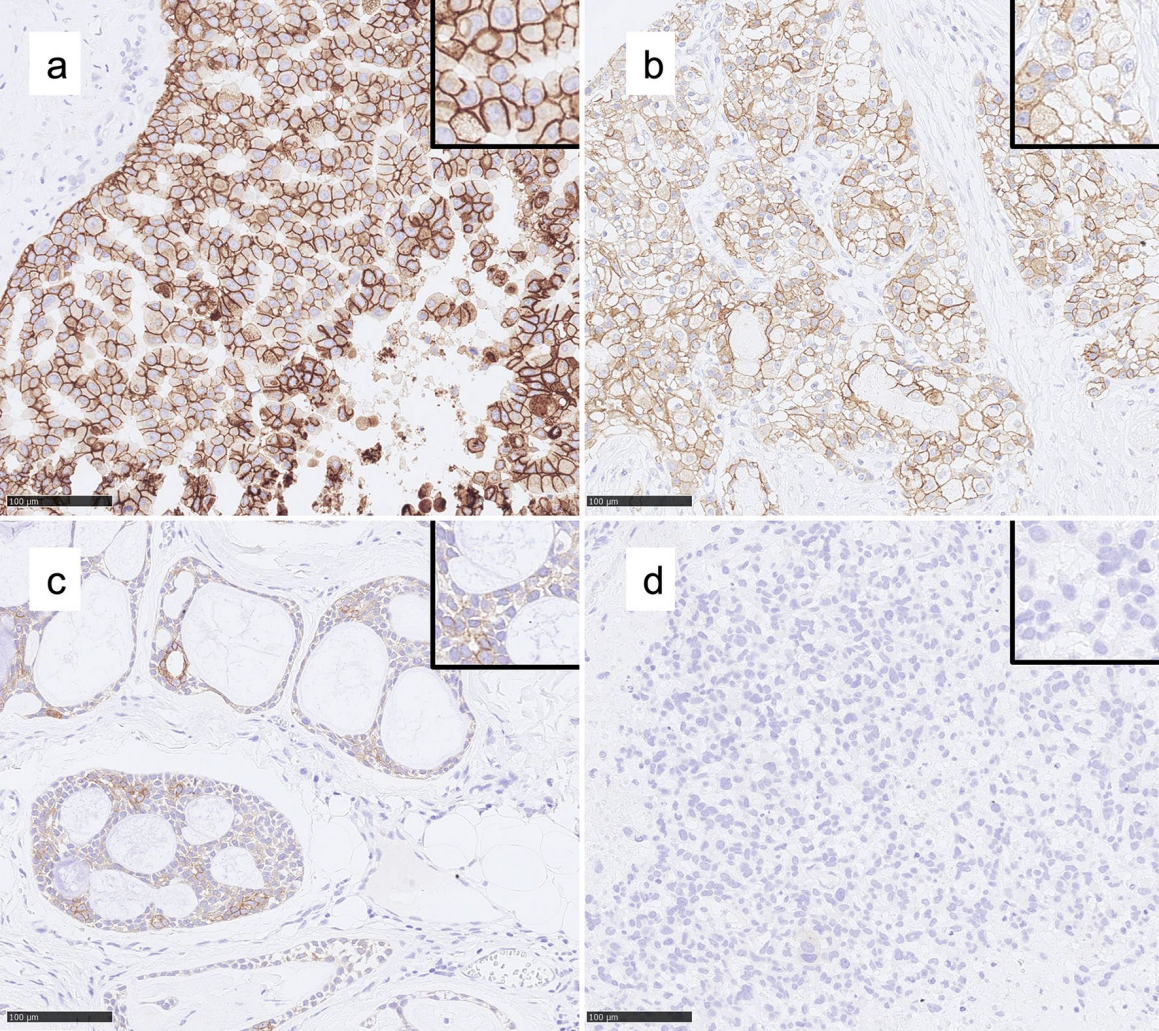
Exemplary IHC demonstrating membranous Trop-2 expression (magnification 200x, insets 400x). **a** High expression in a salivary duct carcinoma. **b** Moderate expression in a salivary duct carcinoma. **c** Weak expression in an adenoid cystic carcinoma. **d** Absent expression in a myoepithelial carcinoma

Out of all 114 SGCs, 50 (43.9%) showed a high, 43 (37.7%) a moderate and 11 (9.6%) a weak expression of Trop-2. In 10 tumors (8.8%) no protein expression was found (Fig. 1). All of the following entities showed a positive Trop-2 protein expression (either weak, moderate or high): mucoepidermoid carcinomas (n = 24, 100%), acinic cell carcinomas (n = 10, 100%), adenocarcinomas NOS (n = 9, 100%), secretory carcinomas (n = 7, 100%), basal cell adenocarcinomas (n = 3, 100%), polymorphic adenocarcinomas (n = 1, 100%) and carcinomas ex pleomorphic adenoma (n = 1, 100%). In addition, 95.5% (21 out of 22) of adenoid cystic carcinomas, 95.7% (22 out of 23) of salivary duct carcinomas and 85.7% (6 out of 7) of epithelial myoepithelial carcinomas stained positive for Trop-2. High expression rates were found in more than 50% of all mucoepidermoid carcinomas (60%, 15 out of 24), adenocarcinomas NOS (55.6%, 5 out of 9) and salivary duct carcinomas (60.9%, 14 out of 23). All carcinosarcomas (n = 2), myoepithelial carcinomas (n = 2), undifferentiated carcinomas (n = 1) and oncocytic cell carcinomas (n = 2) showed no Trop-2 expression.

The intra-tumoral range of heterogeneity of Trop-2 expression exceeded one in only 10.5% of cases with high concordance among the different entities, indicating a high homogeneity of Trop-2 expression. Moreover, 20 full sections were stained with Trop-2 (five with negative, two with weak, eight with moderate and five with high expression in the TMA sections). We observed that all five tumors that were assigned “high” displayed a strong staining in 90% of the tumor cells. Full sections from tumors with weak or moderate Trop-2 expression confirmed the initial classification with a homogenous expression pattern. Three well to moderately differentiated carcinomas that were assigned “negative” remained completely negative on full sections. Conversely, an undifferentiated carcinoma that has been classified as “negative” revealed a weak staining pattern in a third of the tumor surface. Another initially “negative” tumor, a de-differentiated salivary duct carcinoma was completely negative in the poorly differentiated component but highly positive for Trop-2 (“high”) in the well-differentiated part.

No statistically significant difference in Trop-2 expression was found between the most prevalent SGC entities (adenoid cystic carcinoma, mucoepidermoid carcinoma, acinic cell carcinoma, adenocarcinoma NOS, salivary duct carcinoma, epithelial myoepithelial carcinoma and secretory carcinoma; p = 0.462). Moreover, Trop-2 expression showed no correlation with tumor localization (parotid gland vs. submandibular gland; p = 0.32), sex (p = 0.744), presence of nodal metastases (p = 0.721), perineural invasion (p = 0.707), vascular invasion (p = 0.576), lymphatic invasion (p = 1.000). Regarding T-stage, there was a trend towards smaller tumors showing Trop-2 expression (p = 0.051).

**Fig. 2:**
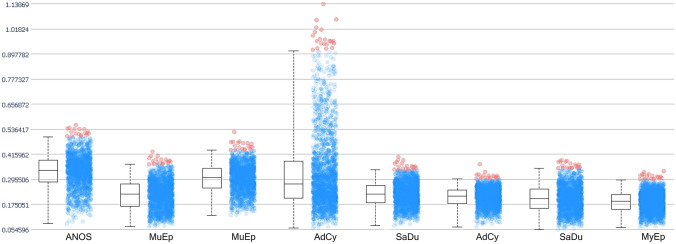
Trop-2 expression in MALDI-MS imaging. Box plot comparing the mean intensity of the m/z value of all detected Trop-2 peptide masses in TMA samples. *ANOS* adenocarcinoma not-otherwise-specified, *MuEp* mucoepidermoid carcinoma, *AdCy* adenoid cystic carcinoma, *SaDu* salivary duct carcinoma, *MyEp* myoepithelial carcinoma

For validation of the immunohistochemistry data, MALDI-MS imaging was performed on 10 exemplary samples. Out of the putative tryptic peptides from Trop-2, seven could be detected. Trop-2 protein was detected in 80% of the samples (Fig. 2). One of the Trop-2 positive samples showed no expression of Trop-2 protein in IHC. The highest intensities of Trop-2 expression in MALDI-MS imaging were in one adenoid cystic carcinoma, one mucoepidermoid carcinoma and one adenocarcinoma NOS. Out of these samples, two showed weak and one strong Trop-2 expression in IHC. Out of the two samples with no Trop-2 expression in MALDI-MS imaging, one mucoepidermoid carcinoma showed moderate expression and one adenoid cystic carcinoma showed no expression in IHC.

### Survival

After a median follow-up of 52 months, the total 5-year disease free survival (DFS) rate was 76.1% (86 out of 114). The lowest 5-year DFS rate was found for adenocarcinoma NOS (50%) and the highest for secretory carcinoma (100%) and acinic cell carcinoma (90%). Trop-2 expression showed no correlation with 5-year DFS (log rank test, p = 0.225). 5-year DFS rate was significantly better for patients in T-stage 1 and 2 (89.3%) compared to T-stage 3 and 4 (61.8%; log rank test, p = 0.001). Lymphatic invasion was found to be negatively correlated with DFS (83.3% for L0 vs. 46.2% for L1; p = 0.001). No correlation was found for sex (p = 0.712).

## Discussion

This is the first study to determine the protein expression of Trop-2 using different techniques in salivary gland carcinomas. Trop-2 is a transmembrane protein with an extracellular domain and its expression is upregulated in tumors cells relative to normal cells [23]. It is involved in several molecular pathways associated with cancer development and has multiple binding partners. Trop-2 expression can increase the expression of Ki-67 causing Ca2 + mobilization from internal stores.

MAPK signaling is activated by Trop-2 leading to increased levels of phosphorylated ERK1 and ERK2 [17]. Activation of ERK can putatively induce proteasomal degradation of FOXO3a and thereby inhibit cell death [31]. Also, MAPK signaling and cell cycle progression are further stimulated by Ca2 + . Trop-2 expression can increase levels of cyclin D1 and cyclin E, helping to mediate ERK1/2 cell cycle progression. Subsequently, ERK signaling induces AP-1 transcription factor which plays a crucial role in the regulation of tumor-associated target genes during carcinogenesis [32]. AP-1 can induce angiogenesis via VEGF (vascular endothelial growth factor), activate cell proliferation via cyclins and CDKs and cause cell invasion and metastasis via matrix metalloproteinases, podoplanin, Ezrin and CD44. Furthermore, AP-1 can induce epithelial to mesenchymal transition via podoplanin allowing for the nuclear translocation of β-catenin, eventually causing cell growth via downstream effectors [17].

Trop-2 overexpression has been described in a variety of malignant tumors, such as in colorectal, esophageal, lung, ovarian or head and neck cancer [17]. Several clinical trials have been investigating Trop-2 as a potential target. PF-06664178, an antibody–drug conjugate delivering an auristatin microtubule inhibitor, was tested in a phase I study in patients with advanced therapy-resistant solid tumors. Eleven out of 31 patients showed stable disease and no complete or partial response was observed [33]. DS-1062a, an antibody–drug conjugate delivering a topoisomerase I inhibitor and derivate of exatecan, is currently being tested for the treatment of unresectable advanced and incurable NSCLC in an ongoing first in human study [23]. Sacituzumab govitecan (or IMMU-132), an antibody–drug conjugate comprising the active metabolite of irinotecan (SN-38) conjugated to an anti-Trop-2 antibody, has been investigated in several trials. In a phase I study in patients with platinum-resistant urothelial carcinoma, it was well tolerated and three out of six patients had a clinically significant response, with a progression free survival of 6.7–8.2 months [34]. Due to these results, a phase II trial has been initiated. For metastatic NSCLC patients, Sacituzumab govitecan was tested in a phase II study with 54 pretreated patients. A clinical benefit rate, defined as complete or partial response or stable disease for more than four months was observed in 43% of the patients. The median response duration to therapy was six months, with a median overall survival of 9.5 months and median progression free survival of 5.2 months [35]. For SCLC patients, Sacituzumab govitecan has also been evaluated in a phase II study. In 60% of the patients tumor shrinkage was observed and a clinical benefit rate was reported for 34% with a median overall survival of 7.5 months and progression free survival of 3.7 months [25]. However, the most promising results have been achieved for triple-negative breast cancer. Bardia et al. have recently investigated the use of sacituzumab govitecan in 108 cases of refractory triple-negative breast cancer. They reported a response rate of 33.3%, including a complete response in 2.8%, with a median duration of response of 7.7 months and a median progression free survival of 5.5 months [26]. These results have led to the approval of sacituzumab govitecan (TRODELVY, Immunomedics, Inc.) by the U.S. Food and Drug Administration for adult patients with metastatic triple-negative breast cancer who received at least two prior therapies for metastatic disease.

In the current study, among the 91.2% of SGCs with Trop-2 expression, 81.6% exhibited a high to moderate protein concentration in immunohistochemistry. More than half of the cohort consisted of adenoid cystic, mucoepidermoid and salivary duct carcinomas. Thus, the most prevalent tumor entities are well represented in the collective. The rates of Trop-2 expression were particularly high in adenoid cystic carcinomas, salivary duct carcinomas, epithelial myoepithelial carcinomas and mucoepidermoid carcinomas. The results of IHC were validated with MALDI-MS Imaging on 10 exemplary samples. Trop-2 protein expression was detected in 80%. There were two discordant findings regarding Trop-2 expression. One sample showed negative Trop-2 expression in IHC but protein expression in MALDI-MS imaging, and another with negative Trop-2 expression in MALDI-MS imaging showed moderate expression in IHC. A possible explanation for this could be the fact that consecutive TMA sections were used. We have also considered the heterogeneity of protein expression within the tumor. For this purpose, we analyzed all tumors with four tumor biopsies from different tumor sites in TMA format and additionally 20 tumors on full tumor sections. Most of the tumors (89.5%) showed a homogeneous expression of Trop-2. Carcinomas with a high Trop-2 pattern were homogeneously positive even on full section, while undifferentiated or dedifferentiated carcinomas displayed a more heterogeneous Trop-2 expression. This underlines the significance of our TMA-based analysis and allows conclusions to be drawn about the potential therapeutic effectiveness of sacituzumab govitecan in SGCs. The more homogeneously a therapy-relevant biomarker is expressed in the tumor, the more likely it is to be effectively targeted.

In comparably designed studies (e.g., Gatsby study), in which a biomarker (Her2/neu)—also determined on the tumor cells—was combined with an antibody–drug conjugate (trastuzumab emtansine) specifically directed against the biomarker, the very heterogeneous distribution of Her2/neu in gastric cancer was a possible relevant reason for the failure of this study [36]. The same drug combination is effective in breast cancer, possibly because (in contrast to gastric cancer) breast cancer usually shows a very homogeneous expression of Her2/neu.

Given the homogeneous expression of Trop-2 in SGC, future clinical studies will have to verify the relationship between Trop-2 protein expression and sacituzumab govitecan. From today's perspective, however, SGCs seem to be good candidates for a Trop-2-targeted therapy.

In our cohort, Trop-2 expression showed no correlation with the presence of nodal metastases, perineural, vascular or lymphatic invasion and 5-year disease free survival. This is in contrast to the findings of a meta-analysis by Ping et al., who reported a significant association of Trop-2 overexpression and short disease free survival in different solid tumors [24]. Therefore, the role of Trop-2 as a prognostic biomarker in SGCs remains inconclusive.

Limitations of our study could be the retrospective fashion of our analyses. The limited number of cases among each individual tumor type make it difficult to establish correlations with clinical parameters or outcome. However, due to the low incidence of certain SGC subtypes, desirable prospective studies with sufficient participants are particularly challenging. Nevertheless, despite these shortcomings we are convinced that the results of this exploratory study can be followed up in clinical studies with Trop-2 as a target in salivary gland carcinomas.

In summary, we were able to demonstrate that several types of salivary gland carcinoma immunohistochemically express Trop-2 with variable intensity. Since there are currently few systemic treatment options for advanced SGCs, Trop-2 represents a promising target for further clinical studies, such as with sacituzumab govitecan.

**Table 1:** Overview of all included samples organized by histologic group

	All (n = 114)	AdCy (n = 22)	MuEp (n = 24)	ACC (n = 10)	ANOS (n = 9)	SaDu (n = 23)	EpMy (n = 7)	SecC (n = 7)	OTH (n = 12)
Localization (gland)									
Parotid	103 (90.4)	16 (72.7)	24 (100)	10 (100)	9 (100)	20 (87)	7 (100)	6 (85.7)	11 (91.7)
Submandibular	11 (9.6)	6 (27.3)	0	0	0	3 (13)	0	1 (14.3)	1 (8.3)
Demographics									
Female	59 (51.8)	13 (59.1)	18 (75)	5 (50)	5 (55.6)	6 (26.1)	2 (28.6)	3 (42.9)	7 (58.3)
Male	55 (48.2)	9 (40.9)	6 (25)	5 (50)	4 (44.4)	17 (73.9)	5 (71.4)	4 (57.2)	5 (41.7)
Age	55.8 ± 17.9	51.5 ± 14.25	42.5 ± 16.9	51.4 ± 18.6	63.9 ± 15.6	66 ± 11.5	61 ± 18.8	48.6 ± 20	68 ± 11.1
Clinical parameter									
T1-2	57 (50)	10 (45.5)	15 (60)	5 (50)	6 (66.7)	7 (30.4)	4 (57.2)	5 (71.4)	5 (41.7)
T3-4	55 (48.2)	11 (50)	8 (33.3)	5 (50)	3 (33.3)	16 (69.6)	3 (42.8)	2 (28.6)	7 (58.3)
N/A	2 (1.8)	1 (4.5)	1 (6.7)	0	0	0	0	0	0
N +	35 (30.7)	7 (31.8)	3 (12.5)	1 (10)	3 (33.3)	20 (87)	0	1 (14.3)	0
N/A	4 (3.5)	1 (4.5)	1 (4.2)	1 (10)	0	0	0	0	1 (8.3)
Pn1	38 (33.3)	9 (40.9)	2 (16.7)	2 (20)	4 (44.4)	17 (73.9)	0	1 (14.3)	3 (25)
N/A	11 (9.6)	4 (18.2)	2 (16.7)	1 (10)	0	2 (8.7)	1 (14.3)	0	1 (8.3)
V1	9 (7.9)	0	2 (8.3)	0	3 (33.3)	2 (8.7)	0	0	2 (16.7)
N/A	11 (9.6)	6 (27.3)	1 (4.2)	1 (10)	0	2 (8.7)	0	1 (14.3)	0
L1	13 (11.4)	0	1 (4.2)	1 (10)	1 (1.1)	9 (39.1)	0	0	1 (8.3)
N/A	10 (8.8)	6 (27.3)	1 (4.2)	1 (10)	0	2 (8.7)	0	0	0
Trop-2 expression									
Negative	10 (8.8)	1 (4.5)	0	0	0	1 (4.3)	1 (14.3)	0	7 (58.3)
Low	11 (9.6)	3 (13.6)	0	2 (20)	3 (33.3)	0	1 (14.3)	0	2 (16.7)
Moderate	43 (37.7)	12 (54.5)	9	5 (50)	1 (11.1)	8 (34.8)	3 (42.9)	4 (57.2)	1 (8.3)
High	50 (43.9)	6 (27.4)	15	3 (30)	5 (55.6)	14 (60.9)	2 (28.6)	3 (42.9)	2 (16.7)

Percentages per entity are given in brackets, means are reported ± standard deviation for metric data. *AdCy* adenoid cystic carcinoma, *MuEp* mucoepidermoid carcinoma, *ACC* acinic cell carcinoma, *ANOS* adenocarcinoma not otherwise specified; *SaDu* salivary duct carcinoma, *EpMy* epithelial-myoepithelial carcinoma, *SecC* secretory carcinoma, *OTH* others, including polymorphic adenocarcinoma (n = 1); basal cell carcinoma (n = 3), myoepithelial carcinoma (n = 2), carcinoma ex pleomorphic adenoma (n = 1), carcinosarcoma (n = 2), poorly differentiated carcinoma (n = 1), oncocytic cell carcinoma (n = 2); N/A: information not available; Pn1: perineural invasion; V1: vascular invasion; L1: lymphatic invasion
